# Celiac Patient With New Episodes of Diarrhea: A Case Report

**DOI:** 10.7759/cureus.32092

**Published:** 2022-12-01

**Authors:** Joana Dias Antunes, Ivo Barreiro, Luísa Loureiro, Abílio Gonçalves

**Affiliations:** 1 Internal Medicine, Hospital Distrital da Figueira da Foz, Figueira da Foz, PRT

**Keywords:** auto immune, celiac disease, autoimmune diseases, chronic diarrhea, lymphocytic colitis

## Abstract

Celiac disease is a relatively common autoimmune disease that affects the gut's ability to process gluten. It is frequently associated with other autoimmune diseases.

In this article, the authors present a clinical case of a 65-year-old female patient with a history of celiac disease and autoimmune hypothyroidism. This patient was admitted to the emergency room with generalized edema and chronic diarrhea with mucus as well as reports of unusual weight loss. A requested fecal analysis tested positive for fecal calprotectin. An endoscopic study further displayed flattening of the intestinal villi. A subsequent biopsy expressed overlapping evidence for both celiac disease and lymphocytic colitis.

This case illustrates how a diagnosis of microscopic colitis should be explored when celiac patients with a history of a stable gluten-free diet display a sudden onset of chronic diarrhea. As the symptoms associated with this disease can often become debilitating, an early diagnosis and treatment are crucial.

## Introduction

Celiac disease (CD) is a common autoimmune disease that, in the last few decades, is being diagnosed at an increasingly earlier stage, especially in Europe, North America, and Oceania [1]. However, CD appears to be relatively uncommon in both Southeast Asia and Sub-Saharan Africa [1]. Across the world, the average overall prevalence of CD in the population is around 1%, with the prevalence being twice as high in females [1].

CD is an immune-mediated enteropathy, precipitated by gluten in the diet, which primarily affects the small intestine and induces a chronic inflammatory state, with a consequent decrease in nutrient absorption and secretion of water and solutes [2]. The classic presentation is characterized by abdominal pain, chronic watery diarrhea, steatorrhea, and nutritional deficits. Extraintestinal manifestations are common and include chronic fatigue and anemia [1].

The gold standard method for its diagnosis is the proximal small bowel mucosal biopsy. The demonstration of the presence of enteropathy in the small bowel biopsy, such as villous atrophy, cryptic hyperplasia, and intraepithelial lymphocytosis, confirms the diagnosis [1]. Testing the presence of antibodies (anti-transglutaminase, anti-gliadin, and anti-endomysium) is recommended in CD screening as these antibodies have high sensitivity and specificity. However, if such antibody testing is negative but clinical suspicion is high, then a biopsy should be performed [1].

The mainstay of treatment is a gluten-free diet, which improves symptoms and prevents complications. Most patients experience remission of disease activity by excluding gluten from their diets. However, untreated symptomatic disease is associated with both high morbidity and mortality. CD is associated with other autoimmune diseases, namely Graves' disease, Hashimoto's thyroiditis, and type 1 diabetes mellitus [2]. A strong association with microscopic colitis has also been described.

## Case presentation

A 65-year-old female patient came to the emergency department due to unexplained weight loss, generalized edema, and chronic diarrhea with mucus. She reported complaints of watery diarrhea, stool with mucus, and unexplained weight loss (19%) within two months of evolution. She had a personal history of CD, arterial hypertension, and autoimmune thyroiditis. She was medicated with furosemide 40 mg once a day (qd) and cloxazolam 2 mg qd. The patient denied tobacco consumption, introduction of new drugs, and recent long-haul trips. She reported sporadic alcohol consumption in a social context.

On objective examination, she was oriented and cooperative, hemodynamically stable, apyretic, and emaciated. She presented marked abdominal distension, pain on superficial and deep palpation, in all quadrants, without peritoneal irritation, and had a sign of an ascitic wave present.

The analytical study revealed anemia, thrombocytosis, severe hypoalbuminemia, increased transaminases, alkaline phosphatase, gamma-glutamyl transpeptidase, and increased international normalized ratio. There were no changes in renal function or inflammatory parameters (Table 1). She was admitted to study chronic diarrhea and unexplained weight loss. Several diagnostic hypotheses were put forward, such as non-compliance with therapy (gluten-free diet), alcoholism, and lymphoma/colon neoplasm.

**Table 1:** Analytical study carried out on admission

Blood Study	Normal Range	Results
Leukocytes	4-10.5 x 10^3^/uL	10.4 x 10^3^/uL
Neutrophils		69.5%
Hemoglobin	11.5-16.5 g/dL	11.1 g/dL
Platelets	150-450 x 10^3^/uL	644 x 10^3^/uL
International normalized ratio		1.8
Glutamic-oxaloacetic transaminase	0-32 U/L	40 U/L
Glutamic pyruvic transaminase	0-31 U/L	62 U/L
Alkaline phosphatase	35-104 U/L	187 U/L
Total bilirubin	<1 mg/dL	0.36 mg/dL
Gamma-glutamyl transferase	5-36 U/L	52 U/L
Total proteins	6.4-8.3 g/dL	4.5 g/dL
Albumin	3.4-4.8 g/dL	1.4 g/dL
Lactate dehydrogenase	240-480 U/L	502 U/L
Creatinine	0.5-0.9 mg/dL	0.5 mg/dL
Sodium	136-145 mEq/L	133 mEq/L
Potassium	3.5-5.1 mEq/L	3.4 mEq/L
C-reactive protein	<5 mg/L	3.24 mg/L
Sedimentation rate	<20 mm/h	13 mm/h
Procalcitonin	<0.5 ng/mL	0.64 ng/mL
Proteins in urine 24 h	28-141 mg/day	172 mg/day
Thyroid hormones		Normal
Anti-thyroglobulin antibody	<60 UI/mL	187 U/mL
IgA	70-400 mg/dL	218 mg/dL
IgG	700-1600 mg/dL	769 mg/dL
IgM	40-230 mg/dL	208 mg/dL
Hepatitis C, B, and HIV serology		Negative

Albumin replacement was started, with progressive improvement of edema. A stool study was requested and fecal calprotectin was positive (Tables 1, 2). Thoraco-abdomino-pelvic computed tomography revealed marked ascites and diffuse interstitial edema of the subcutaneous plane that suggests anasarca.

**Table 2:** Stool study carried out on admission

Stool Study	
Fecal calprotectin (normal range < 50 mg/kg)	92 mg/Kg
Leukocyte research	Negative
Stool cultures	Negative
Parasitological exam	Negative
Clostridium difficile toxin A and B research	Negative

**Figure 1:**
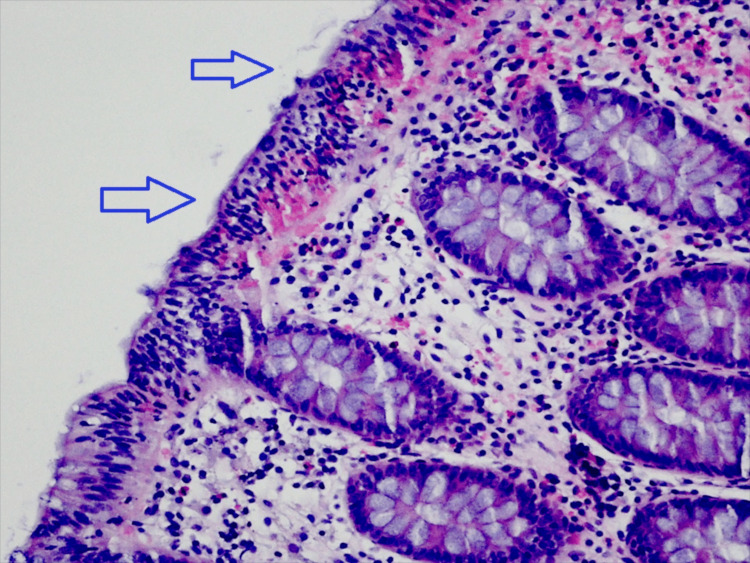
Histological sample of colonic mucosa biopsy (enlarged) Colonic mucosa shows infiltration of the epithelium by multiple lymphocytes (arrows). Hematoxylin and eosin, 100x.

**Figure 2:**
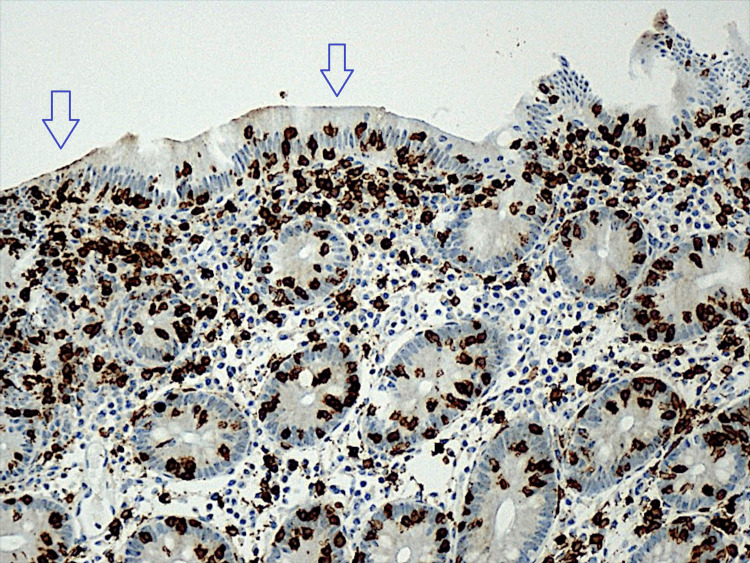
Second histological sample of colonic mucosa biopsy (enlarged) Staining for CD3 in the colonic mucosa shows an evident increase in the number of intraepithelial lymphocytes (arrows). Horseradish peroxidase-diaminobenzidine (HRP-DAB), 100x.

An upper digestive endoscopy was performed, which did not show relevant alterations. A rectosigmoidoscopy was also performed which showed mucosa without apparent changes. Biopsies were performed according to clinical information. The results revealed the criteria for lymphocytic colitis (Figures 1, 2).

The patient was discharged with budesonide 9 mg qd and a recommendation for an indication of a reinforced gluten-free diet. She was referred for internal medicine and gastroenterology consultation. She started progressive weaning from budesonide two months after discharge, and it was stopped after five months without complications. CD was also controlled with a gluten-free diet, and the initial weight was regained. She is currently no longer in therapy and is being observed every six months in a gastroenterology consultation.

## Discussion

Microscopic colitis is a chronic inflammatory disease of the colon and includes two entities: lymphocytic colitis and collagenous colitis. The colon looks normal in a colonoscopy, so the diagnosis depends on a biopsy of the colonic mucosa [1,2]. Histologically, lymphocytic colitis is characterized by enlargement of the lamina propria, infiltration of chronic inflammatory cells, and recruitment of intraepithelial lymphocytes, with damage to the surface of the lamina propria. In the case of collagenous colitis, there is also a thickening of the subepithelial collagen layer [3].

Microscopic colitis is characterized by chronic, watery, and non-bloody diarrhea, urgency, and fecal incontinence. Half of the patients complain of abdominal pain, weight loss, and extraintestinal complaints such as arthralgia, arthritis, or uveitis [2]. Laboratory findings include anemia, high sedimentation rate, and positive autoantibodies in 50% of cases, namely rheumatoid factor, antinuclear, antimitochondrial, and antineutrophilic cytoplasmic antibodies [2].

Treatment is based on the use of antidiarrheal agents and/or corticosteroid therapy. Budesonide is recommended in patients with persistent diarrhea, despite therapy with antidiarrheal agents, at a dose of 9 mg daily, for six to eight weeks [4]. After clinical stabilization, gradual discontinuation is indicated. In uncontrolled patients, therapy should be continued for 12 or more weeks. Prednisone is reserved for patients who do not respond to budesonide [2].

Patients with CD are at an increased risk of microscopic colitis (approximately 70 times higher than that in the general population) [3]. Approximately, 15% of patients with microscopic colitis have CD. However, only 4% of celiac patients have a concomitant diagnosis of lymphocytic or collagenous colitis [3]. Middle-aged women have a higher risk of developing microscopic colitis, with an average age of 65 years at diagnosis [2].

The relationship between the two pathologies seems to be in part genetic: It is known that HLA-DR3-DQ2 predisposes to CD and is also associated with microscopic colitis [4]. Therefore, in celiac patients previously controlled on a gluten-free diet and with an onset of chronic diarrhea, it is essential to consider the concomitant diagnosis of microscopic colitis. If clinical suspicion is high, a colonoscopy with biopsy should be performed to confirm the diagnosis [5].

The clinical follow-up of this patient, in particular, is important as she has a very significant autoimmune background. In addition to autoimmune thyroiditis, CD, and lymphocytic colitis, it brings together all immune-mediated pathologies. The appearance of other autoimmune diseases is a possibility to be taken into account, so the follow-up of this patient in consultation is essential.

## Conclusions

The importance of this clinical case lies in the fact that the appearance of microscopic colitis in celiac patients is uncommon. However, this differential diagnosis should not be forgotten when new episodes of diarrhea appear in previously well-controlled patients on a gluten-free diet. Early diagnosis and treatment are essential to improve the prognosis.

## References

[1] Arasaradnam RP, Brown S, Forbes A (2018). Guidelines for the investigation of chronic diarrhoea in adults: British Society of Gastroenterology, 3rd edition. Gut.

[2] Villanueva MS, Alimi Y (2015). Microscopic colitis (lymphocytic and collagenous), eosinophilic colitis, and celiac disease. Clin Colon Rectal Surg.

[3] Lauret E, Rodrigo L (2013). Celiac disease and autoimmune-associated conditions. Biomed Res Int.

[4] Barta Z, Zold E, Nagy A, Zeher M, Csipo I (2011). Celiac disease and microscopic colitis: a report of 4 cases. World J Gastroenterol.

[5] Stewart M, Andrews CN, Urbanski S, Beck PL, Storr M (2011). The association of coeliac disease and microscopic colitis: a large population-based study. Aliment Pharmacol Ther.

